# Tumor-antigens and immune landscapes identification for prostate adenocarcinoma mRNA vaccine

**DOI:** 10.1186/s12943-021-01452-1

**Published:** 2021-12-06

**Authors:** Xiaonan Zheng, Hang Xu, Xianyanling Yi, Tianyi Zhang, Qiang Wei, Hong Li, Jianzhong Ai

**Affiliations:** 1 Institute of Urology, West China Hospital, Sichuan University, Chengdu, 610041 China; 2 Department of Urology, West China Hospital, Sichuan University, Chengdu, 610041 China; 3 Institute of Systems Genetics, West China Hospital, Sichuan University, Chengdu, 610041 China

**Keywords:** mRNA vaccine, Prostate adenocarcinoma, Immunotherapy, Tumor antigens, Immune subtypes, Immune landscape

## Abstract

Prostate adenocarcinoma (PRAD) is a leading cause of death among men. Messenger ribonucleic acid (mRNA) vaccine presents an attractive approach to achieve satisfactory outcomes; however, tumor antigen screening and vaccination candidates show a bottleneck in this field. We aimed to investigate the tumor antigens for mRNA vaccine development and immune subtypes for choosing appropriate patients for vaccination. We identified eight overexpressed and mutated tumor antigens with poor prognostic value of PRAD, including KLHL17, CPT1B, IQGAP3, LIME1, YJEFN3, KIAA1529, MSH5 and CELSR3. The correlation of those genes with antigen-presenting immune cells were assessed. We further identified three immune subtypes of PRAD (PRAD immune subtype [PIS] 1–3) with distinct clinical, molecular, and cellular characteristics. PIS1 showed better survival and immune cell infiltration, nevertheless, PIS2 and PIS3 showed cold tumor features with poorer prognosis and higher tumor genomic instability. Moreover, these immune subtypes presented distinguished association with immune checkpoints, immunogenic cell death modulators, and prognostic factors of PRAD. Furthermore, immune landscape characterization unraveled the immune heterogeneity among patients with PRAD. To summarize, our study suggests KLHL17, CPT1B, IQGAP3, LIME1, YJEFN3, KIAA1529, MSH5 and CELSR3 are potential antigens for PRAD mRNA vaccine development, and patients in the PIS2 and PIS3 groups are more suitable for vaccination.

**Supplementary Information:**

The online version contains supplementary material available at 10.1186/s12943-021-01452-1.

## Background

Prostate adenocarcinoma (PRAD) is the second diagnosed and the fifth death-related malignancy among men worldwide [1]. Positive responses in patients with PRAD were rarely observed after immunotherapies including programmed cell death protein 1 (PD-1), PD-Ligand 1 (PD-L1), or cytotoxic T lymphocyte antigen 4 (CTLA4). Previously, Sipuleucel-T brought prostate cancer immunotherapy into a sharper focus whereas no significant effect was reported regarding progression-free survival [2, 3]. Therefore, novel therapeutics should be developed for effective PRAD treatment. In the past 2 years, under the background of coronavirus disease-2019 pandemic, the enthusiasm for mRNA vaccine development, showing advantages of flexibility, productivity, non-genomic integration, and low immunogenicity [4], was also brought into the field of cancer therapy [5].

Previous phase I/II clinical trial showed good tolerability and favorable immune activation of mRNA vaccines CV9103 for PRAD; however, the subsequent clinical trials of CV9104 containing two more antigens (prostatic acid phosphatase [PAP] and Mucin-1) were terminated due to failure of improving the overall survival (OS) [6]. These findings indicated that antigen selection is critical for activating antigen-presenting cells (APCs) and immune response. Moreover, the identification of immune subtypes of patients with PRAD for mRNA vaccination is another crucial factor for the curative effect [7]. Hence, this study, exploring novel candidate tumor antigens for PRAD mRNA vaccine and identifying suitable patients for vaccination, aims to pave an avenue for the application of mRNA vaccine in PRAD population.

## Results and discussion

### Identification of potential tumor antigens of PRAD

**Fig. 1:**
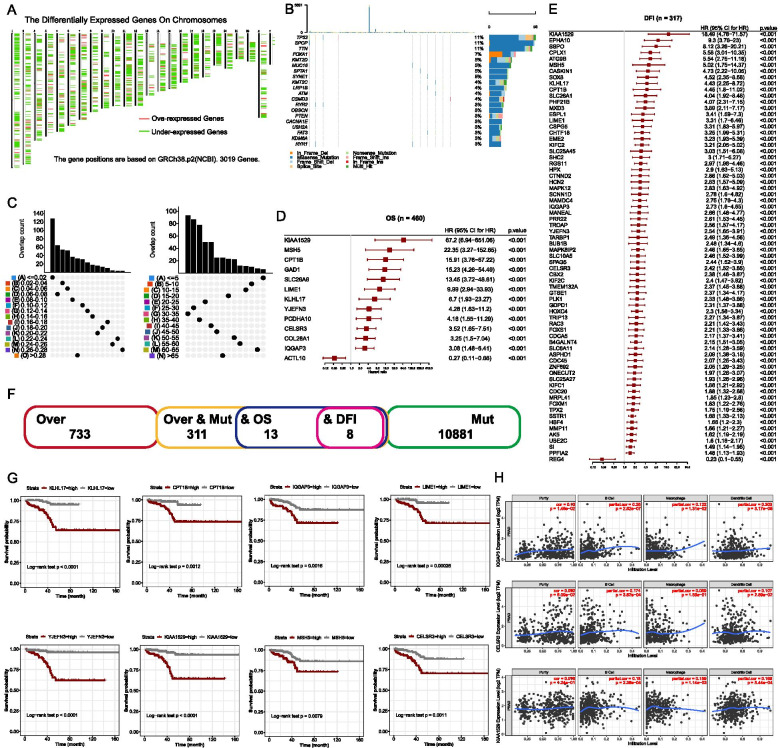
Identification of potential PRAD-specific antigens for mRNA vaccine development. **A** Distribution of the upregulated and downregulated genes across the chromosomes. **B** Mutation status of the top 20 mostly mutated genes of each PRAD sample. **C** Samples overlapping in altered genome fraction and mutation count groups. **D** Potential tumor antigens associated with the overall survival (OS) of PRAD (13 genes). **E** Potential tumor antigens associated with disease-free PRAD interval (70 genes). **F** Identification of tumor antigens associated with both the OS of disease-free PRAD interval (8 genes). **G** Kaplan–Meier survival curves of patients with PRAD according to the expression of eight potential tumor PRAD antigens. **H** Correlation of IQGAP3, CELSR3, and KIAA1529 expression with the infiltration of B cells, macrophages, and dendritic cells

A total of 733 overexpressed genes in TCGA-PRAD samples were identified (Fig. S1A, B) and their distribution in chromosomes was shown in Fig. 1A. We then identified 10881 genes that potentially encode tumor-specific antigens through calculating the fraction of altered genome and tumor mutational counts (Fig. 1B, C). Ten genes with the highest altered genome fractions and mutation counts were displayed in Fig. S1C and D. The 733 overexpressed genes were then intersected with the 10881 mutated tumor antigen-encoding genes, and 311 genes were identified afterwards (Fig. S1E). Cox regression revealed that 13 genes were significantly associated with OS (Fig. 1D) and 70 genes were significantly associated with disease free interval (DFI) (Fig. 1E). Further intersection analysis indicated eight genes, including KLHL17, CPT1B, IQGAP3, LIME1, YJEFN3, MSH5, CELSR3 and KIAA1529, were correlated with both OS and DFI (Fig. 1F). The Kaplan–Meier survival curves of OS for those eight genes were shown in Fig. 1G, and the higher expression of them was indicative of worse survival. The correlation between them and the infiltration of major APCs, including B cells, macrophages as well as dendritic cells (DCs), was also analyzed. Figure 1H showed a significantly positive correlation of IQGAP3, CELSR3, and KIAA1529 with APCs, whereas negative for CPT1B (Fig. S1F). Taken together, our evidence identified tumor-specific antigens owning potentiality of mRNA vaccine development for PRAD.

To the best of our knowledge, our study is the first to systematically screen suitable tumor antigens for mRNA vaccine development in PRAD. Despite the non-application of these antigens in mRNA vaccine development until the present, some of these antigens have been functionally explored in previous studies. For instance, CPT1B silencing could reduce cell proliferation and invasion in PRAD cell lines and its expression level might be regulated by androgen receptors [8]. IQGAP3 was upregulated in most cancer types and predicted a poor prognosis. It might also participate in the Paris forrestii antitumor effect [9]. Besides, CELSR3 downregulation significantly suppresses PRAD cell proliferation and migration [10]. MSH5 has been reported as a pleiotropic susceptibility locus for several cancers and was identified as a novel candidate gene warranting additional follow-up as a prospective PRAD risk locus [11]. However, KLHL17, KIAA1529, LIME1, and YJEFN3 were not fully elucidated in PRAD or other cancers. Their function in cancers especially PRAD warrants further exploration.

### Identification and validation of the PRAD immune subtypes

**Fig. 2:**
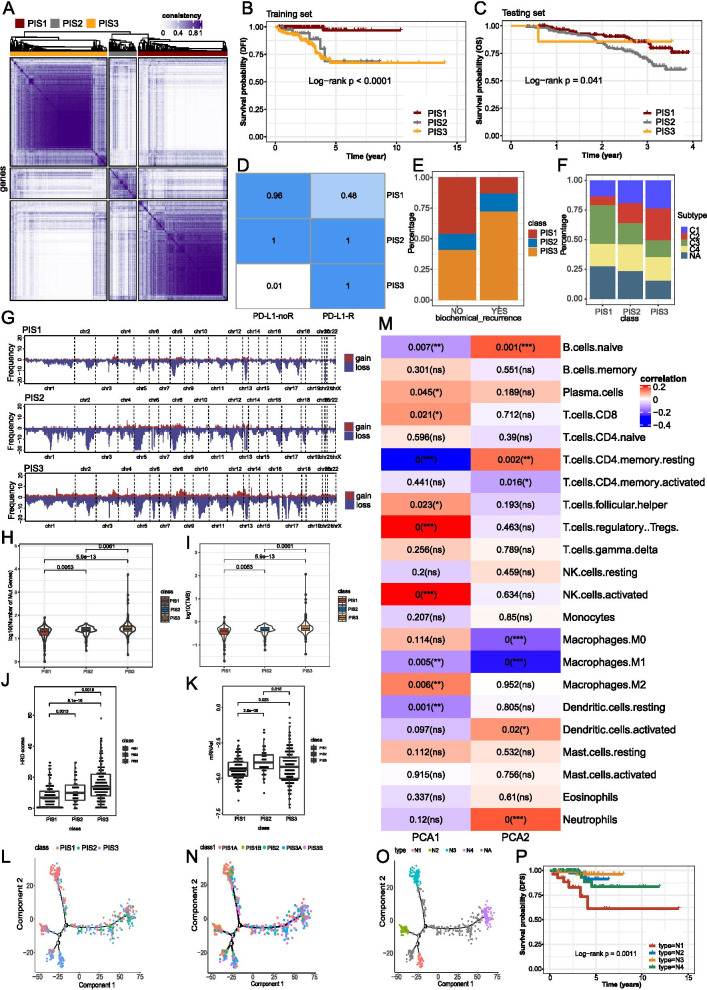
Identification of immune subtypes and immune landscape of PRAD. **A** Identification of the clusters of TCGA-PRAD cohort using partition around medoids algorithm. **B-C** Survival comparison among the PRAD immune subtypes in the training cohort and validation cohort. **D** The prediction of the response to anti-PD-L1 immunotherapy for PRAD immune subtypes. **E** The distribution of PIS1, PIS2, and PIS3 in the groups with or without biochemical recurrence. **F** Association between PRAD immune subtypes and existing pan-cancer immune subtypes. **G** Copy number variation (CNV) across chromosomes across the PRAD immune subtypes. **H-I** Mutation counts and tumor mutation burden across PIS1, PIS2, and PIS3. **J** Homologous recombination deficiency score for each PRAD immune subtype. **K** The comparison of mRNA stemness index across PIS1 to PIS3. **L** Immune landscape of PRAD. Each point represents a patient and the immune subtypes are color-coded. **M** Association between two principal components and immune cells. **N** Immune landscape of the PIS1 and PIS3 subsets. **O** Immune landscape of four subsets of samples from extreme locations. **P** Survival curves of four subsets of samples from extreme locations. * *P* < 0.01, ** *P* < 0.001, and *** *P* < 0.0001

A total of 13426 immunogenic genes were obtained from the MSigDB c7 datasets, 23 of which were associated with predictive survival outcomes through Lasso regression. The PAM algorithm accordingly identified the optimal number of clusters as three based on the training cohort (Fig. 2A). The accumulative curve and delta area of clustering were displayed in Fig. S2A and B. Principal component analysis showed the distribution of TCGA-PRAD individuals in each cluster (Fig. S2C), and heatmap revealed the differential expression of partial immunogenic genes across the three clusters (Fig. S2D). Importantly, the PRAD immune subtype 1 (PIS1) consistently had better survival outcomes compared to the PIS2 and PIS3 in both training cohort (*P* < 0.0001) and validation cohort (*P* = 0.041) (Fig. 2B and S2C).

### Clinical, mutational and immunological features of the PRAD immune subtypes

Clinical features of the PRAD immune subtypes were assessed. The predicted response to immunotherapy of the subtypes indicated that PIS2 and PIS3 were more likely to respond to anti-PD-L1 treatment (Fig. 2D). PIS3 had a higher frequency of biochemical recurrence (Fig. 2E) and pathological N1 stage and higher pathological T stage (Fig. S2E, F). Moreover, patients in PIS3 also had a higher risk of receiving radiation therapy (Fig. S2G). This evidence implies that PIS2 and PIS3 are associated with more aggressive clinical features and more suitable for immunotherapy.

The correlation of existing PRAD biomarkers with PRAD immune subtypes was evaluated. The expression of HOXC6, whose higher expression indicated short survival and higher recurrence rate of PRAD [12], was significantly higher in PIS2 and PIS3 than in PIS1 (Fig. S3A). Nevertheless, PDK4 and STAT3, two classical genes whose low expression was associated with worse survival of PRAD [13, 14], were significantly less expressed in PIS2 and PIS3 (Fig. S3B and C). Notably, our PRAD immune subtypes were also compared with previously published pan-cancer immune subtypes. Figure 2F demonstrated that the C3 subtype had a decreasing distribution, and C1 and C2 showed an increasing tendency across PIS1 to PIS3. Interestingly, C3 was claimed to be a positive marker of prognosis but C1 and C2 were negatively associated with survival [15], which was consistent with our findings.

In terms of mutation status, PIS3 and PIS2 presented more frequent CNV (either gain or loss) across the chromosomes (Figs. 2G and S3D). Similarly, it could be seen from the mutation landscape of the immune subtypes that PIS3 had a more frequent mutation of the top 20 most mutated genes (Fig. S3E), and PIS2 and PIS3 had significantly higher tumor mutation counts (Fig. 2H). In addition, tumor mutation burden (TMB) was also found to be significantly heavier in PIS2 (*P* = 0.0053) and PIS3 (*P* < 0.0001) than in PIS1 (Fig. 2I). Our results also demonstrated that PIS2 and PIS3 had higher telomeric allelic imbalance (HRD-NtAI), large scale transition (HRD-LST) and loss of heterozygosity (HRD-LOH) and combined homologous recombination deficiency (HRD scores) (Fig. 2J, S3F-H). Moreover, mRNA stemness index (mRNAsi) was also higher in PIS2 and PIS3 compared to that of PIS1 (Fig. 2K).

As shown in Fig. S4A and D, the tendency of stromal score, immune score, and tumor purity was variable across the subtypes in both training and validation cohorts. In the training cohort, PIS3 had richer infiltration of M2 macrophages and memory B cells, but PIS1 had more infiltration of naïve B cells and memory-resting CD4+ T cells (Fig. S4B, C). Consistently, PIS3 still had a higher degree of memory B cell infiltration compared to PIS1 and PIS2 in the validation cohort (Fig. S4E, F). Hence, PIS3 implies having a better performance of presenting tumor antigen during the immune response.

The anticancer immune activity of the three immune subtypes was calculated with the TIP analysis. PIS1 performed better at recruiting CD4+ T cells, Th22 cells, and monocytes, whereas PIS2 and PIS3 were still proved to be better at recruiting B cells (Fig. S5A). These outcomes may explain the better survival of PIS1 and also indicate the suitability of PIS2 and PIS3 for receiving tumor vaccines. As for the immune modulators, a total of 37 ICP genes and 25 ICD genes were analyzed, which revealed that 31 ICP genes and 18 ICD genes were differentially expressed across the immune subtypes in the training cohort from TCGA datasets (Fig. S5B, C). Interestingly, fewer ICPs and ICDs were significantly differentially expressed among three clusters in the GEO cohorts, and the PIS3 cluster showed markedly lower ICP and ICD expression (Fig. S5D, E). These findings indicated that the immunotyping showed a distinct expression pattern of ICPs and ICDs, and these modulators could be utilized as potential markers for treatment with mRNA vaccines.

### Immune subtype-based landscape of PRAD

The gene expression value of each patient across the three PRAD immune subtypes was used to build the immune landscape of PRAD (Fig. 2L), with PIS1, PIS2 and PIS3 were generally distributed in different branches of the tree. Principal component 1 (horizontal axis) was positively correlated with plasma cells and M2 macrophages, but negatively correlated with naïve B cells, resting DCs and M1macrophages. Interestingly, principal component 2 (vertical axis) had a positive correlation with DCs and naïve B cells, but a negative correlation with M0 and M1 macrophages (Fig. 2M). The general distribution of PIS1 can be observed to contrast PIS3. Also, individuals within the same immune subtype of PIS1 and PIS3 showed opposing distribution. Therefore, PIS1 and PIS3 were further divided (Fig. 2N), which turned out that PIS1A had a generally higher enrichment score regarding activated DCs and memory B cells compared to PIS1B (Fig. S6A). Similarly, PIS3A had a higher enrichment score of activated DCs, activated B cells and memory B cells than PIS3B (Fig. S6B). Therefore, tumor antigen may be more effective in PIS1A and PIS3A compared to PIS1B and PIS3B, respectively. Besides, individuals with extreme distribution in the immune landscape (Fig. 2O) were taken into further survival analysis. Group N1 was associated with the worst survival and group N3 had the best survival outcomes (*P* = 0.0011) (Fig. 2P). The immune subtype-based landscape can potentially designate the precise mRNA vaccine therapeutics for patients with PRAD by identifying immune components of patients with PRAD and predict survival.

### Weighted immunogenic gene co-expression network of PRAD

WGCNA with a fixed soft threshold of nine was used to construct the immunogenic gene co-expression network of PRAD (Fig. S7A-C). Eventually, 9 co-expression modules were obtained (Fig. S7D, E). Distribution analysis showed that PRAD immune subtypes were differentially distributed in most of the modules (Fig. S7F). The negative prognostic value of the pink (Hazard ratio (HR) 2.30, 95% CI 1.59–3.32), magenta (HR 2.19, 95% CI 1.56–3.08), and purple modules (HR 1.61, 95% CI 1.16–2.24) were presented in Fig. S8A. The biological function of the prognostic modules, including B cell activation, and regulation of adaptive immune response were also displayed in Fig. S8B–D. 62 hub genes in the prognostic modules were identified, and three of them (CDC20, ESPL1, MAPK8IP3) were eventually selected after multivariate Cox regression (Fig. S8E, F). Patients were divided into the high-risk and low-risk groups. KM curve demonstrated that the high-risk group had worse survival (*P* = 0.0011) and the area under the receiver operating curve (AUC) was 0.852, indicating a good accuracy of the model (Fig. S8G, H). Thus, this risk model based on immunogenic genes co-expression network may work as a novel biomarker for predicting the prognosis.

### DEG-based risk model construction

391 DEGs across PRAD immune subtypes were found and displayed (Fig. S9A, B). The prognostic value of these DEGs was calculated and 93 DEGs were prognostic. Lasso regression reduced the dimension of these DEGs and 21 genes were finally used to construct the risk model (Fig. S9C–F). The risk of each patient was calculated based on the expression value of the 21 genes and their coefficients in Lasso regression (Fig. S9G, H). Patients were categorized into high-risk or low-risk groups, and the high-risk group had worse survival with an AUC of 0.892 (Fig. S10A, B). Consistently, Fig. S10C and D summarized that PIS2 and PIS3 had higher risk scores with more PIS1 were distributed in the low-risk group, which in reverse proved the accuracy of PRAD immune subtype in predicting PRAD prognosis.

## Conclusions

In this study, KLHL17, CPT1B, IQGAP3, LIME1, YJEFN3, KIAA1529, MSH5 and CELSR3 were identified as potential tumor-specific antigens for PRAD mRNA vaccine development. PRAD patients of PIS2 and PIS3 might be suitable candidates of vaccination. These findings provided new sights in selecting antigens and populations for future PRAD mRNA vaccine development and application.

### Methods and availability of supporting data

Methods and materials used in our study are attached as Supplementary information. All data are freely available from the public databases and the other necessary and reasonable information could be obtained from the corresponding author.

## Supplementary Information

**Additional file 1: Figure S1.** Identification of overexpressed and mutated genes. **A**. Identification of upregulated and downregulated genes in PRAD compared to normal samples. **B**. Heatmap of the expression of upregulated and downregulated genes in the tumor and normal samples. **C.** Top 10 genes with the highest altered genome fractions in the PRAD samples. **D**. Top 10 genes with the highest mutation count in the PRAD samples. **E**. Intersection of the upregulated genes in PRAD and antigen-encoding genes (311 genes). **F**. Correlation of KLHL17, CPT1B, LIME1, YJEFN3, and MSH5 expression with the infiltration of B cells, macrophages, and dendritic cells.

**Additional file 2: Figure S2.** Clustering of PRAD samples and the clinical features of PRAD immune subtypes. Cumulative curve (**A**) and delta area (**B**) of clustering. **C**. Principal component analysis of the distribution of each individual in three clusters in the training cohort. **D**. The differential expression of 23 prognostic immunogenic genes across the three clusters. The distribution of PIS1, PIS2, and PIS3 in the groups diagnosed with different pathologic T stages (**E**) or N stages (**F**) or treated with radiation therapy (**G**).

**Additional file 3: Figure S3.** Correlation of PRAD immune subtypes with existing biomarkers and homologous recombination deficiency score. Differential expression of HOXC6 (**A**), PDK4 (**B**), and STAT3 (**C**) across the PRAD immune subtypes. **D.** Copy number variation (CNV) counts across the PRAD immune subtypes. **E**. Mutation frequency of the top 20 mostly mutated genes in each PRAD immune subtype. **F-H**. Telomeric allelic imbalance score, large scale transition score, and loss of heterozygosity score for each PRAD immune subtype. * *P* < 0.01, ** *P* < 0.001, and *** *P* < 0.0001.

**Additional file 4: Figure S4.** Correlation between the PRAD immune subtypes and the infiltration of immune cells. The comparison of the stromal score, immune score, tumor purity, and immune cells infiltration across PIS1 to PIS3 in the CGA-PRAD cohort (**A-C**) and validation cohort (**D–F**). * *P* < 0.01 and *** *P* < 0.0001.

**Additional file 5: Figure S5.** Immune status of the PRAD immune subtypes. **A**. Association of anticancer immune activity and PRAD immune subtypes. Immune-checkpoint genes and immunogenic cell death genes are differentially expressed across the PRAD immune subtypes in the training (**B–C**) and validation (**D–E**) cohorts. * *P* < 0.01, ** *P* < 0.001, and *** *P* < 0.0001.

**Additional file 6: Figure S6.** Comparison between the subsets of PIS1 and PIS3. **A**. Enrichment score of immune cells between PIS1A and PIS1B. **B**. Enrichment score of immune cells between PIS3A and PIS3B, respectively.

**Additional file 7: Figure S7.** Immune status of the PRAD immune subtypes. Tumor mutation burden (**A**) and mutation counts (**B**) across PIS1, PIS2, and PIS3. **C**. Mutation frequency of the top 20 mostly mutated genes in each PRAD immune subtype. Copy number variation (CNV) across chromosomes (**D**) and CNV count (**E**) in the PRAD immune subtypes.

**Additional file 8: Figure S8.** Identification of hub gene-based risk model of PRAD. **A**. The prognostic value of the co-expression modules. **B–D**. Biological function and signaling pathways that the three prognostic co-expression modules (pink, magenta, and purple) were involved. **E**. Risk score of each PRAD individual. **F**. Differential expression of ESPL1, CDC20, and MAPK8IP3 between the high-risk and low-risk groups. **G**. Survival probability of the high-risk and low-risk groups. **H**. Accuracy of the risk model presented with receiver operating curve.

**Additional file 9: Figure S9.** Construction of differentially expressed genes (DEGs)-based risk model. **A**. Intersection of DEGs between PIS1, PIS2, and PIS3. **B**. Differential expression of DEGs in PIS1, PIS2, and PIS3, and their association with clinical features. **C**. Prognostic value of 21 DEGs that were selected to construct the risk model. **D**. Part of the survival curves of the 21 DEGs. **E–F**. Partial likelihood deviance and coefficients response status of constructing the risk model. **G**. Coefficients of each DEG in the Lasso regression. **H**. Risk classification of each PRAD individual.

**Additional file 10: Figure S10.** Association between PRAD immune subtypes and risk model. **A**. Risk model survival curve. **B**. Risk model receiver operating curve. **C**. Risk score of each PRAD immune subtype. **D**. Distribution of risk groups across the PRAD immune subtypes.

**Additional file 11.**

## Abbreviations

PRAD: Prostate adenocarcinoma
mRNA: Messenger ribonucleic acid
TCGA: The Cancer Genome Atlas
GEO: Gene Expression Omnibus
KLHL17: Kelch Like Family Member 17
CPT1B: Carnitine Palmitoyltransferase 1B
IQGAP3: IQ Motif Containing GTPase Activating Protein 3
LIME1: Lck Interacting Transmembrane Adaptor 1
YJEFN3: YjeF N-Terminal Domain Containing 3
MSH5: MutS Homolog 5
CELSR3: Cadherin EGF LAG seven-pass G-type receptor 3
PIS: PRAD immune subtype
PD-1: Programmed cell death protein 1
PD-L: Programmed cell death protein ligand 1
CTLA4: Cytotoxic T lymphocyte antigen 4
CRPC: Castration-resistant prostate cancer
OS: Overall survival
APCs: Antigen-presenting cells
DFI: Disease-free interval
ICI: Immune-checkpoint inhibitors
TIP: Tracking Tumor Immunophenotype
TIMER: Tumor IMmune Estimation Resource
PAM: Partition around medoids
MSigDB: The Molecular Signatures Database
TMB: Tumor mutation burden
CNV: Copy number variation
HRD: Homologous recombination deficiency
NtAI: Allelic imbalance
LST: Large scale transition
LOH: Loss of heterozygosity
mRNAsi: mRNA stemness index
ICP: Immune-checkpoint
ICD: Immunogenic cell death
WGCNA: Weighted correlation network analysis
DEG: Differential expression gene
DCs: Dendritic cells
HOXC6: Homeobox C6
PDK4: Pyruvate Dehydrogenase Kinase 4
STAT3: Signal Transducer and Activator of Transcription 3
HR: Hazard ratio
CDC20: Cell Division Cycle 20
ESPL1: Extra Spindle Pole Bodies Like 1
MAPK8IP3: Mitogen-Activated Protein Kinase 8 Interacting Protein 3
AUC: Area under the curve
ICB: Immune-checkpoint blockade
