# Supplementary figures and images for: Tumor-antigens and immune landscapes identification for prostate adenocarcinoma mRNA vaccine
Source: Mol Cancer. 2021 Dec 6;20:160. doi: 10.1186/s12943-021-01452-1 (PMC8645679; doi:10.1186/s12943-021-01452-1)

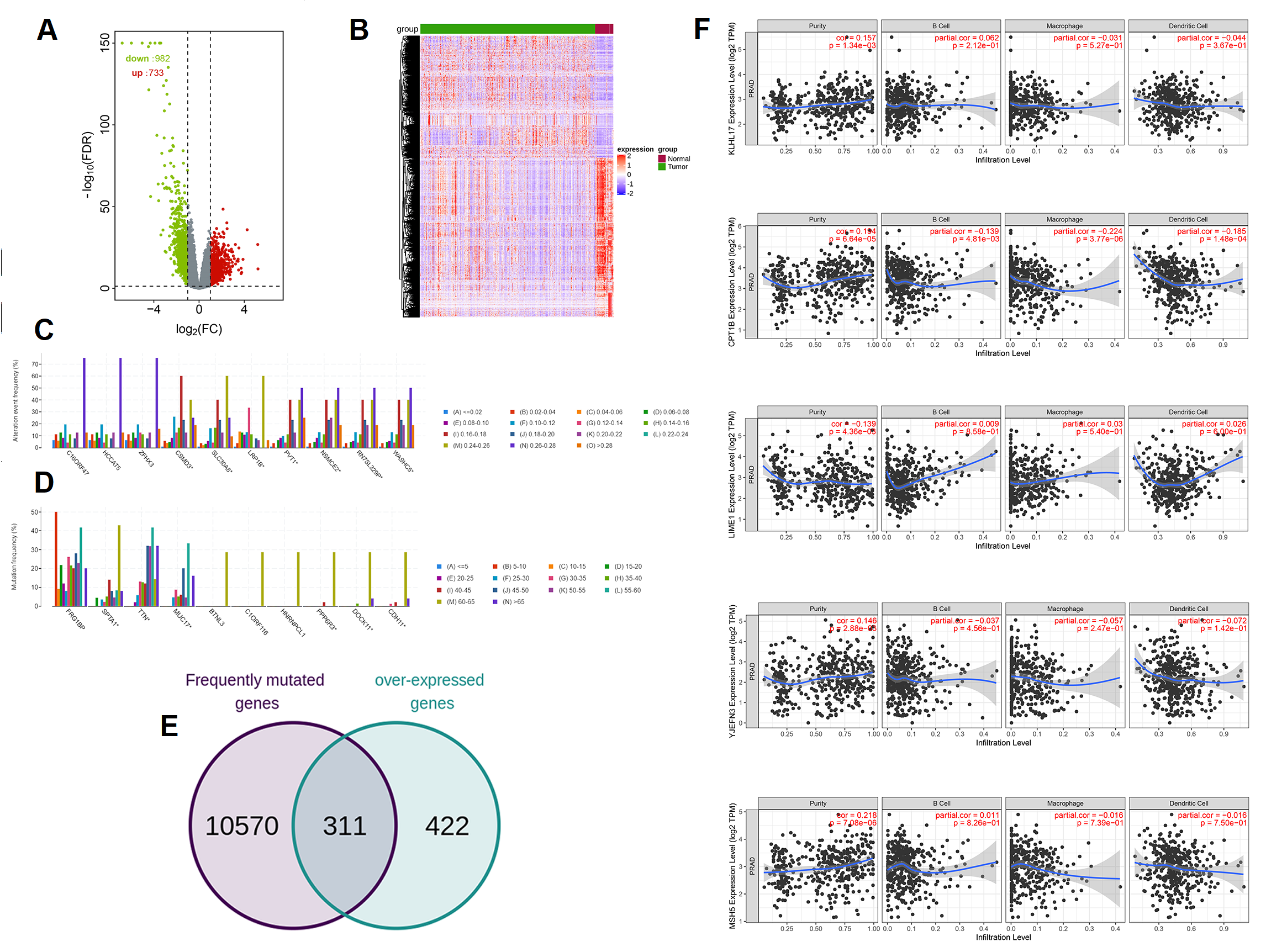

Supplement: Supplementary file 1 — Additional file 1: Figure S1. Identification of overexpressed and mutated genes. A. Identification of upregulated and downregulated genes in PRAD compared to normal samples. B. Heatmap of the expression of upregulated and downregulated genes in the tumor and normal samples. C. Top 10 genes with the highest altered genome fractions in the PRAD samples. D. Top 10 genes with the highest mutation count in the PRAD samples. E. Intersection of the upregulated genes in PRAD and antigen-encoding genes (311 genes). F. Correlation of KLHL17, CPT1B, LIME1, YJEFN3, and MSH5 expression with the infiltration of B cells, macrophages, and dendritic cells. [file 12943_2021_1452_MOESM1_ESM.tif]

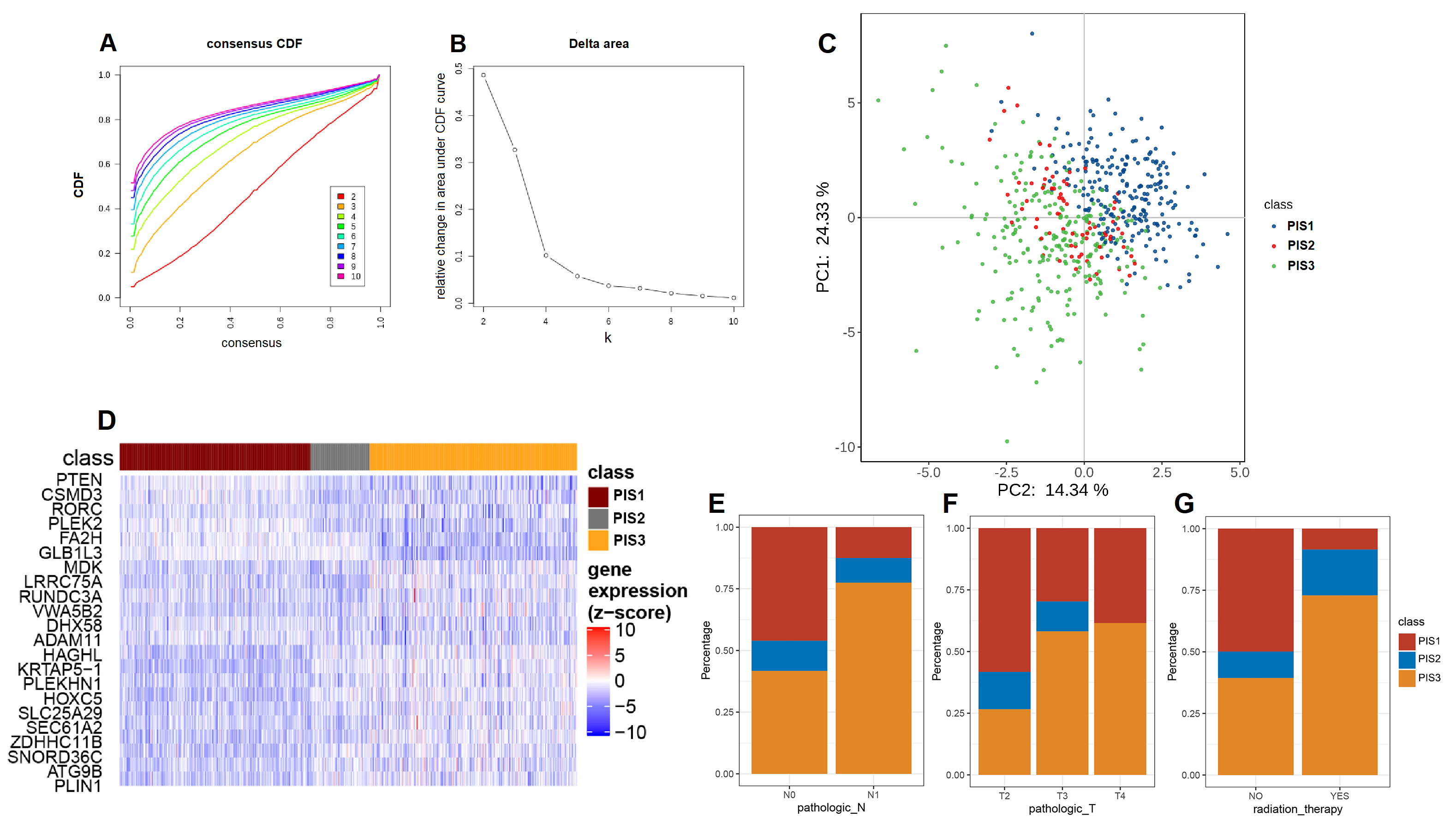

Supplement: Supplementary file 2 — Additional file 2: Figure S2. Clustering of PRAD samples and the clinical features of PRAD immune subtypes. Cumulative curve (A) and delta area (B) of clustering. C. Principal component analysis of the distribution of each individual in three clusters in the training cohort. D. The differential expression of 23 prognostic immunogenic genes across the three clusters. The distribution of PIS1, PIS2, and PIS3 in the groups diagnosed with different pathologic T stages (E) or N stages (F) or treated with radiation therapy (G). [file 12943_2021_1452_MOESM2_ESM.tif]

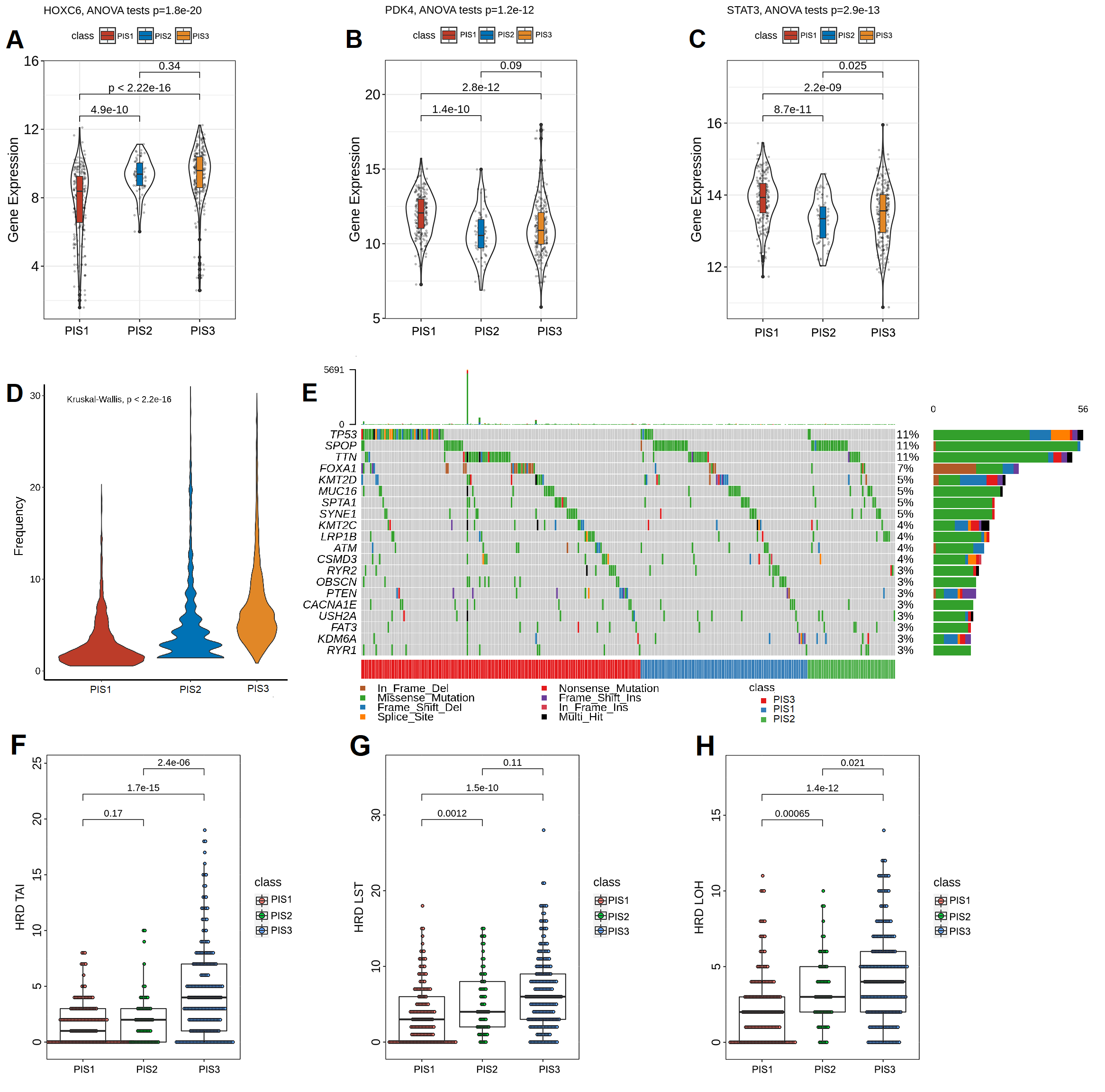

Supplement: Supplementary file 3 — Additional file 3: Figure S3. Correlation of PRAD immune subtypes with existing biomarkers and homologous recombination deficiency score. Differential expression of HOXC6 (A), PDK4 (B), and STAT3 (C) across the PRAD immune subtypes. D. Copy number variation (CNV) counts across the PRAD immune subtypes. E. Mutation frequency of the top 20 mostly mutated genes in each PRAD immune subtype. F-H. Telomeric allelic imbalance score, large scale transition score, and loss of heterozygosity score for each PRAD immune subtype. * P < 0.01, ** P < 0.001, and *** P < 0.0001. [file 12943_2021_1452_MOESM3_ESM.tif]

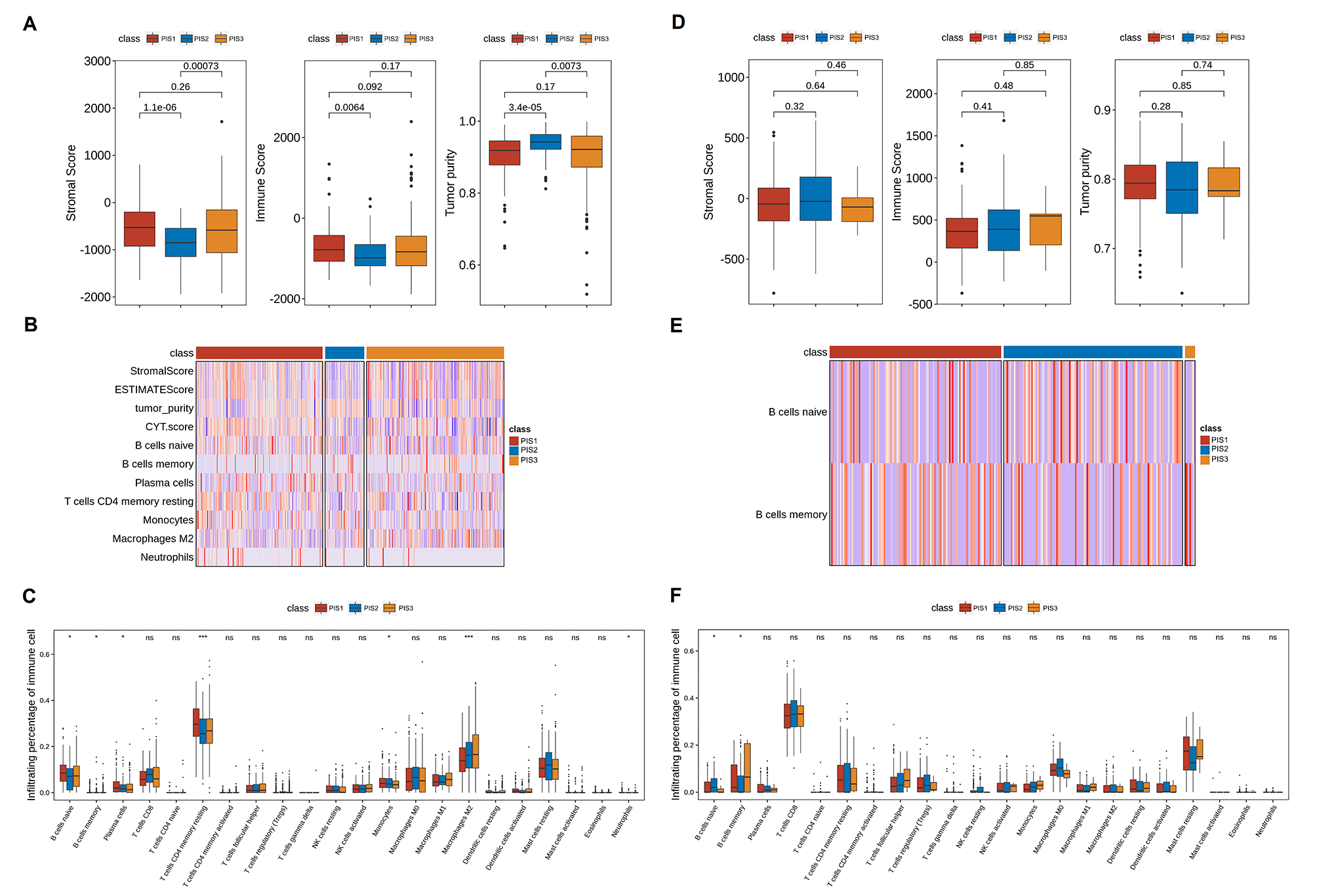

Supplement: Supplementary file 4 — Additional file 4: Figure S4. Correlation between the PRAD immune subtypes and the infiltration of immune cells. The comparison of the stromal score, immune score, tumor purity, and immune cells infiltration across PIS1 to PIS3 in the CGA-PRAD cohort (A-C) and validation cohort (D–F). * P < 0.01 and *** P < 0.0001. [file 12943_2021_1452_MOESM4_ESM.tif]

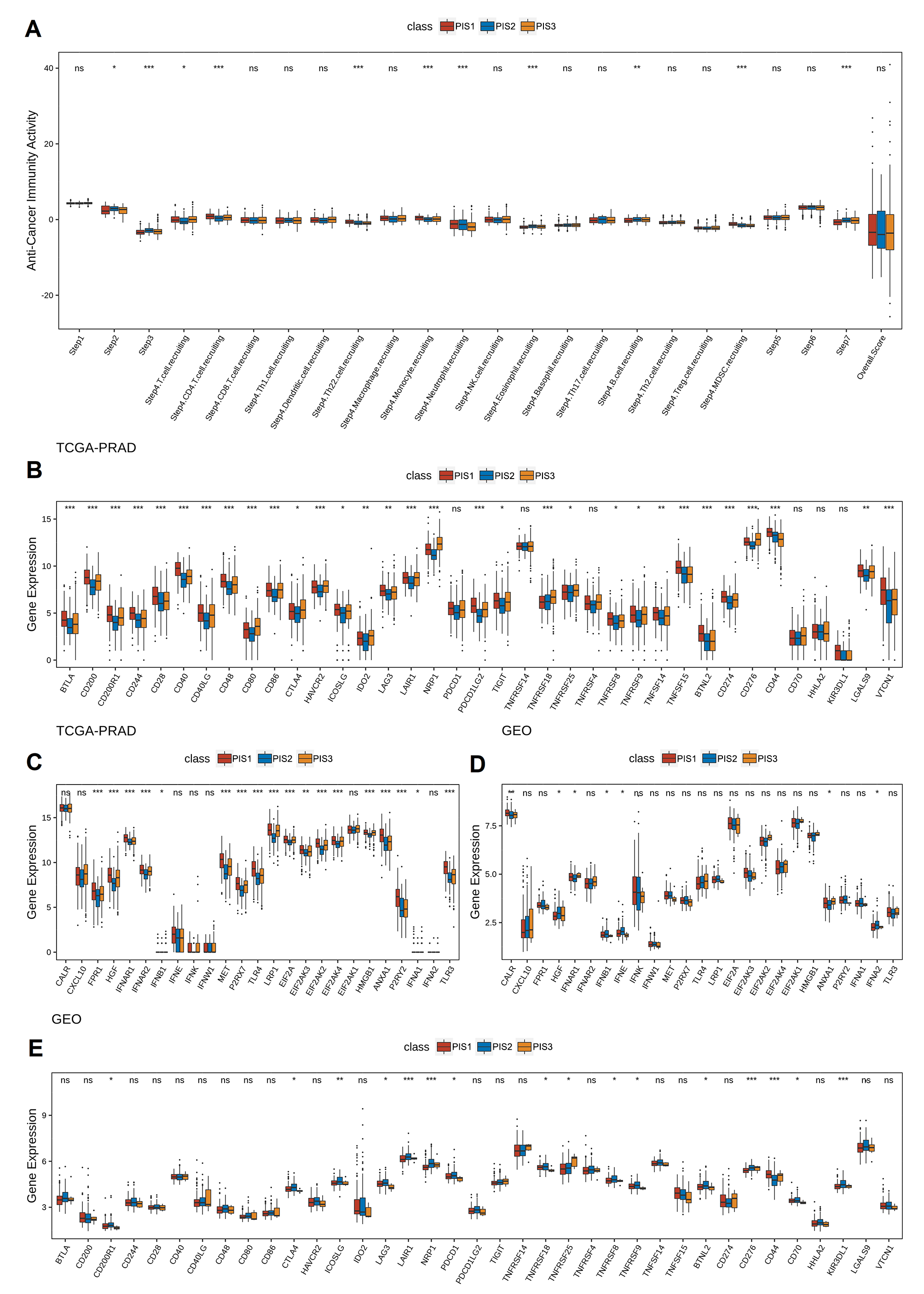

Supplement: Supplementary file 5 — Additional file 5: Figure S5. Immune status of the PRAD immune subtypes. A. Association of anticancer immune activity and PRAD immune subtypes. Immune-checkpoint genes and immunogenic cell death genes are differentially expressed across the PRAD immune subtypes in the training (B–C) and validation (D–E) cohorts. * P < 0.01, ** P < 0.001, and *** P < 0.0001. [file 12943_2021_1452_MOESM5_ESM.tif]

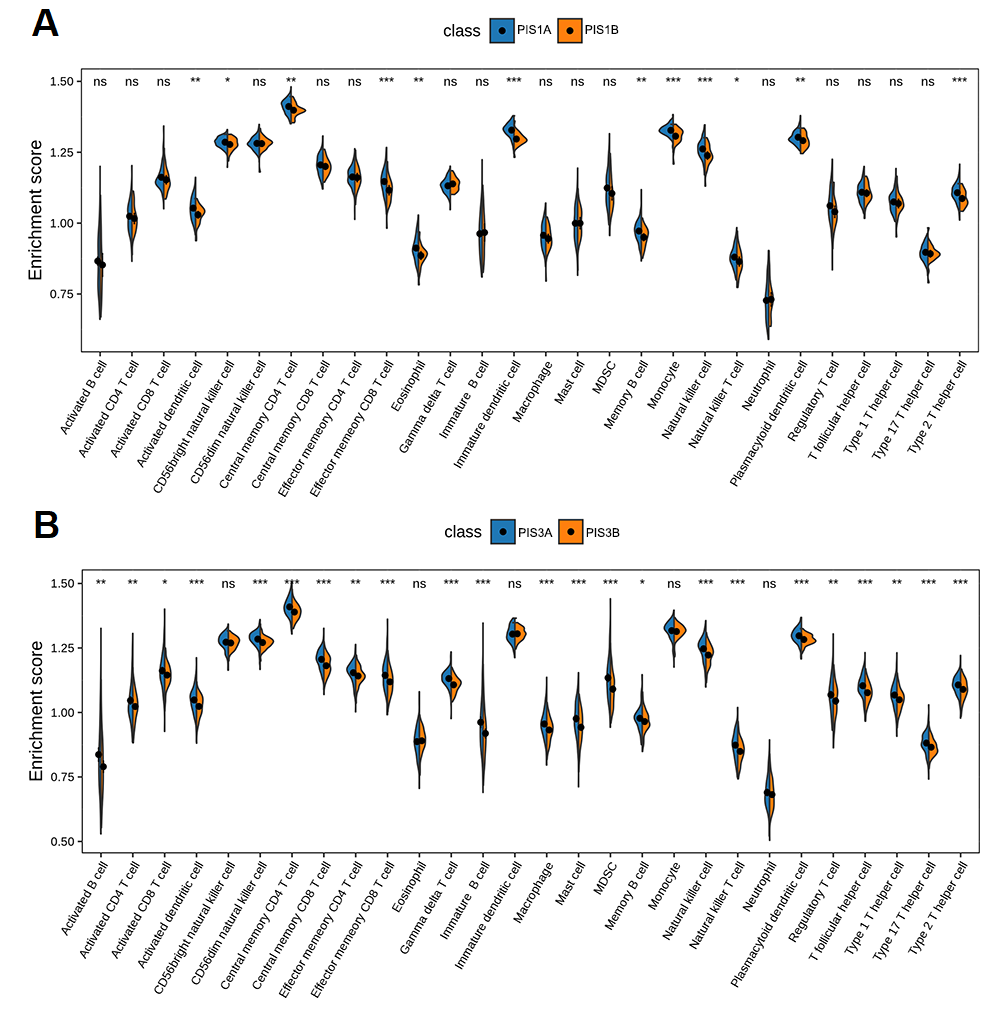

Supplement: Supplementary file 6 — Additional file 6: Figure S6. Comparison between the subsets of PIS1 and PIS3. A. Enrichment score of immune cells between PIS1A and PIS1B. B. Enrichment score of immune cells between PIS3A and PIS3B, respectively. [file 12943_2021_1452_MOESM6_ESM.tif]

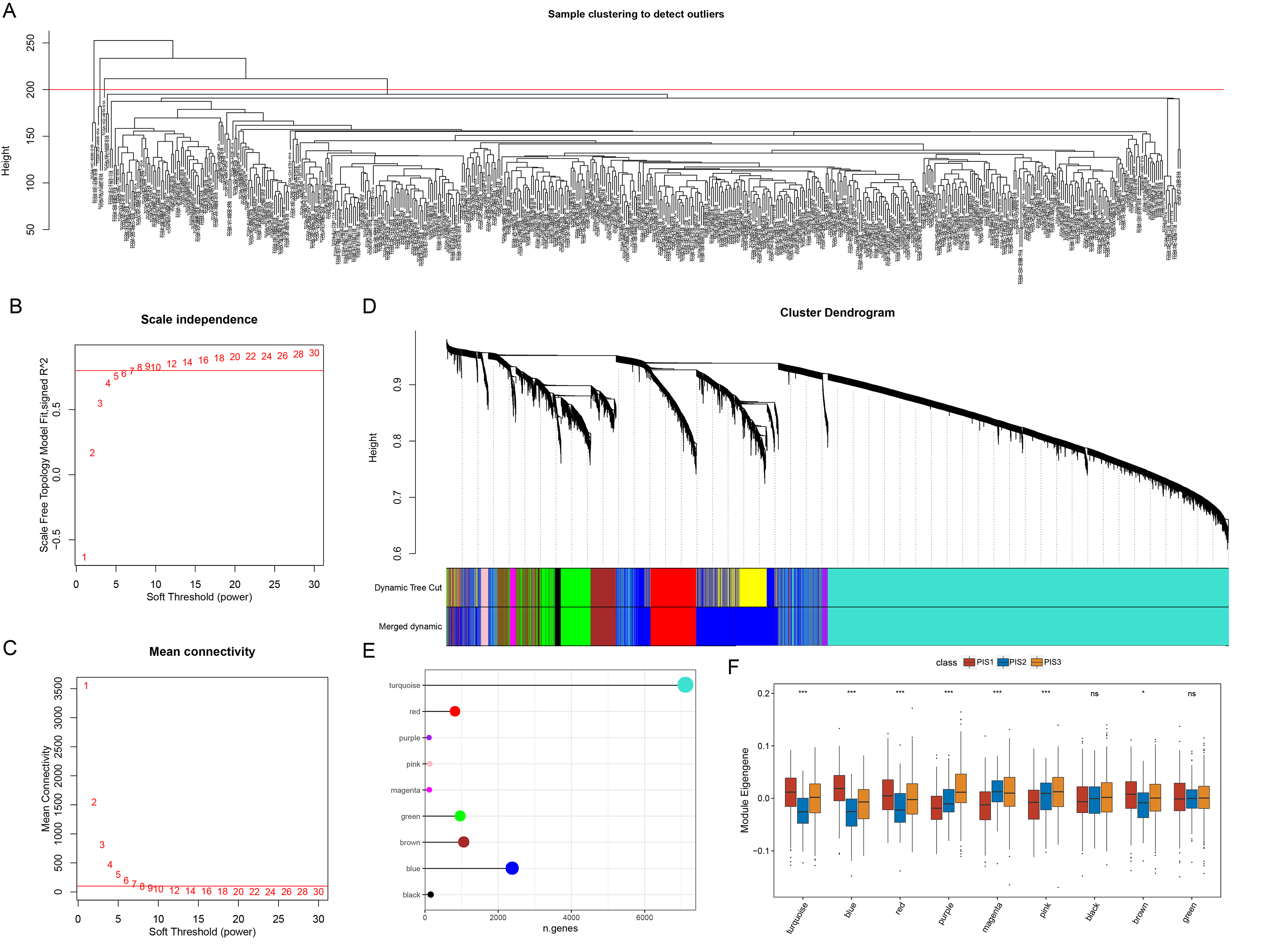

Supplement: Supplementary file 7 — Additional file 7: Figure S7. Immune status of the PRAD immune subtypes. Tumor mutation burden (A) and mutation counts (B) across PIS1, PIS2, and PIS3. C. Mutation frequency of the top 20 mostly mutated genes in each PRAD immune subtype. Copy number variation (CNV) across chromosomes (D) and CNV count (E) in the PRAD immune subtypes. [file 12943_2021_1452_MOESM7_ESM.tif]

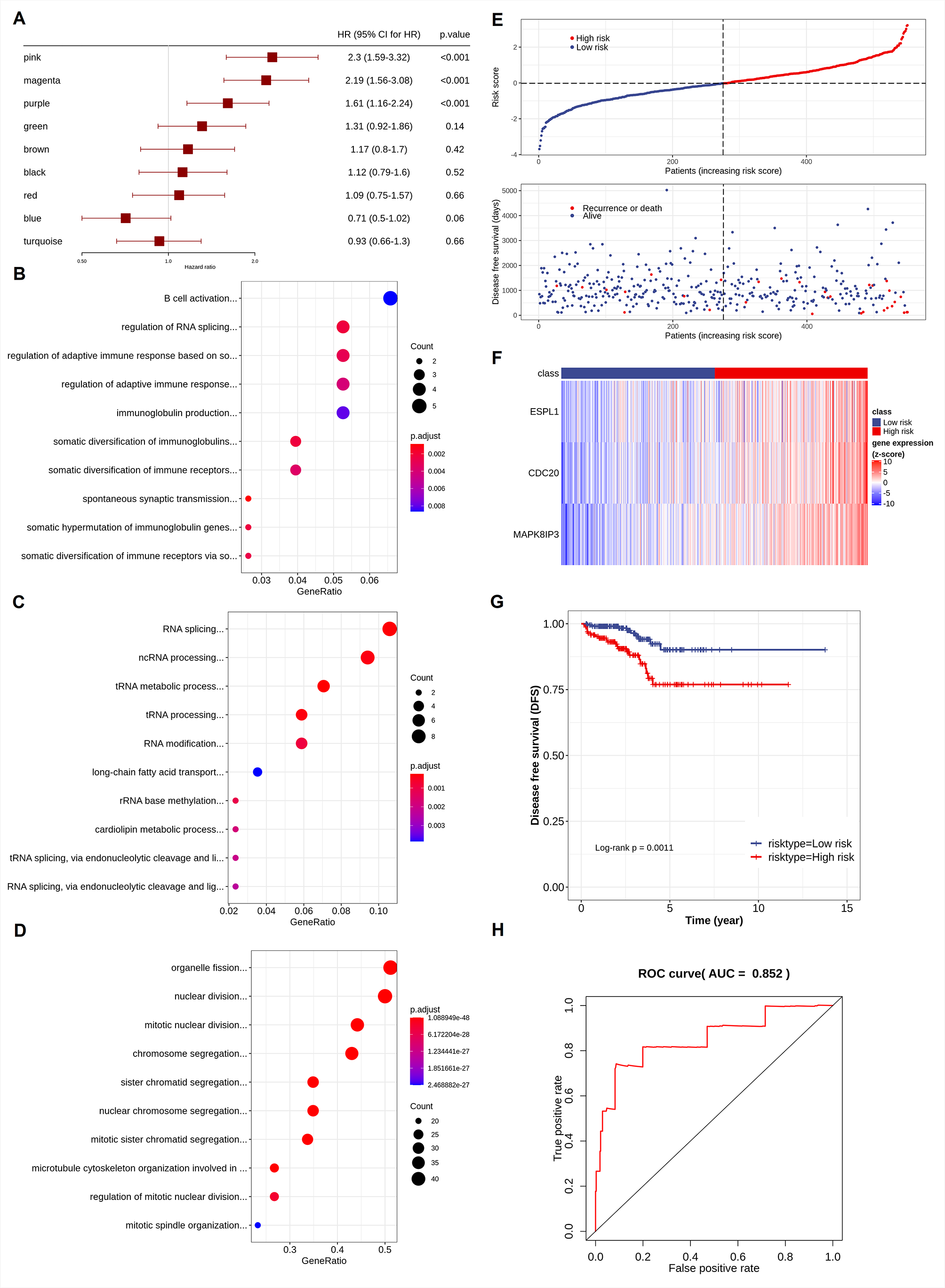

Supplement: Supplementary file 8 — Additional file 8: Figure S8. Identification of hub gene-based risk model of PRAD. A. The prognostic value of the co-expression modules. B–D. Biological function and signaling pathways that the three prognostic co-expression modules (pink, magenta, and purple) were involved. E. Risk score of each PRAD individual. F. Differential expression of ESPL1, CDC20, and MAPK8IP3 between the high-risk and low-risk groups. G. Survival probability of the high-risk and low-risk groups. H. Accuracy of the risk model presented with receiver operating curve. [file 12943_2021_1452_MOESM8_ESM.tif]

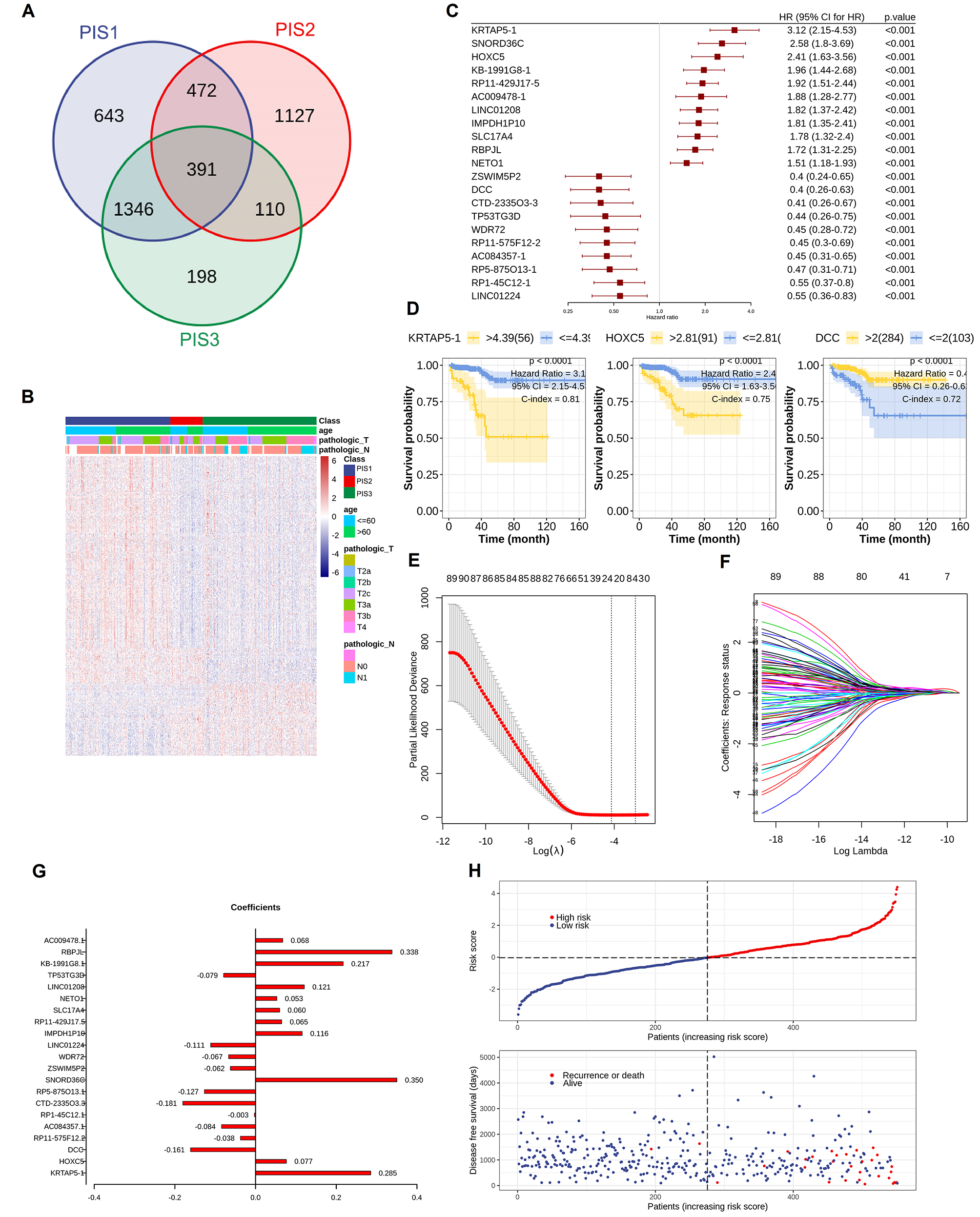

Supplement: Supplementary file 9 — Additional file 9: Figure S9. Construction of differentially expressed genes (DEGs)-based risk model. A. Intersection of DEGs between PIS1, PIS2, and PIS3. B. Differential expression of DEGs in PIS1, PIS2, and PIS3, and their association with clinical features. C. Prognostic value of 21 DEGs that were selected to construct the risk model. D. Part of the survival curves of the 21 DEGs. E–F. Partial likelihood deviance and coefficients response status of constructing the risk model. G. Coefficients of each DEG in the Lasso regression. H. Risk classification of each PRAD individual. [file 12943_2021_1452_MOESM9_ESM.tif]

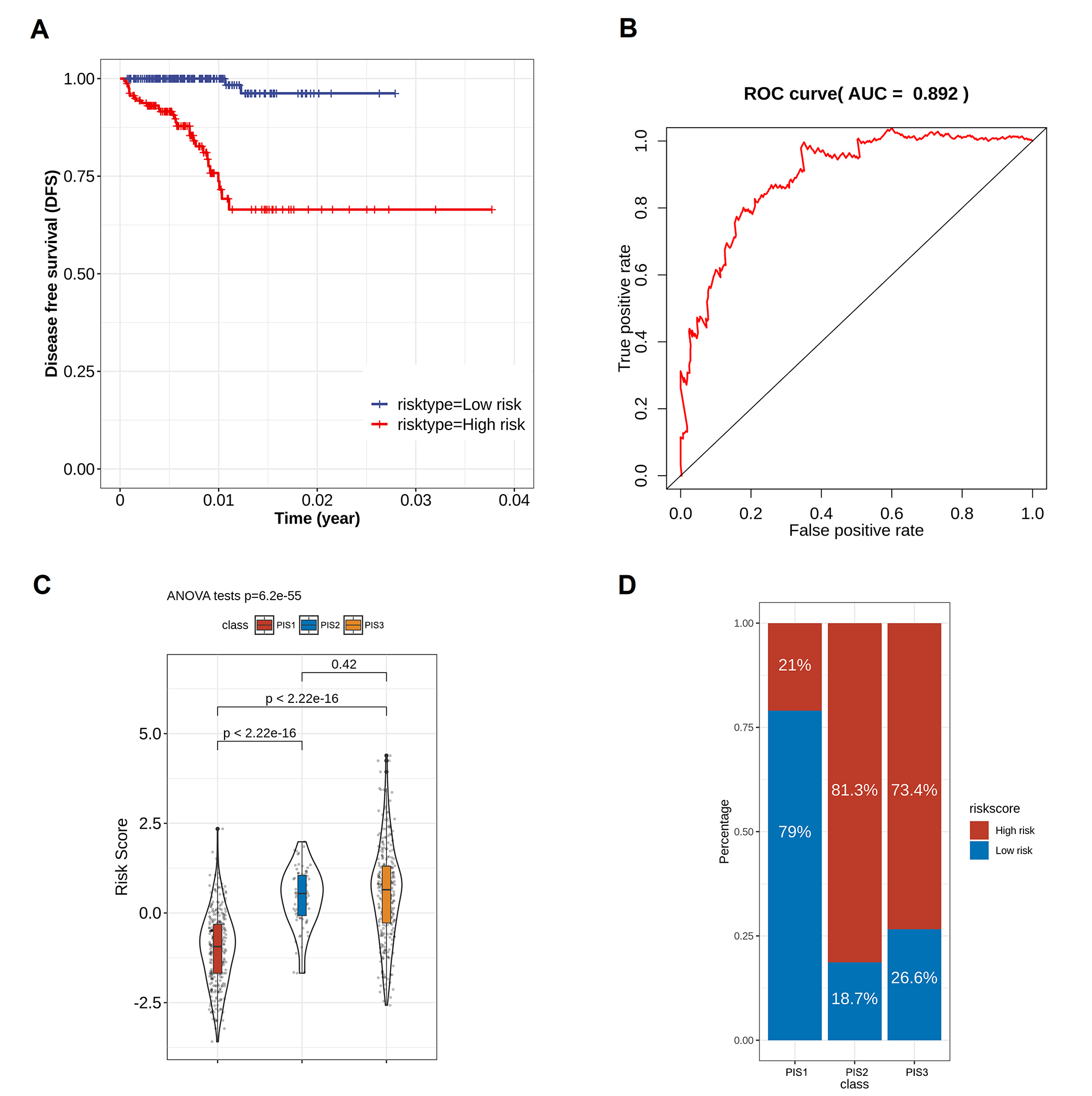

Supplement: Supplementary file 10 — Additional file 10: Figure S10. Association between PRAD immune subtypes and risk model. A. Risk model survival curve. B. Risk model receiver operating curve. C. Risk score of each PRAD immune subtype. D. Distribution of risk groups across the PRAD immune subtypes. [file 12943_2021_1452_MOESM10_ESM.tif]
