# Supplementary material for: Tumor-antigens and immune landscapes identification for prostate adenocarcinoma mRNA vaccine
Source: Mol Cancer. 2021 Dec 6;20:160. doi: 10.1186/s12943-021-01452-1 (PMC8645679; doi:10.1186/s12943-021-01452-1)
Supplement: Supplementary file 11 — Additional file 11. [file 12943_2021_1452_MOESM11_ESM.docx]

**Supplementary Methods and Materials**

**Data preparation and processing**

Gene expression data of The Cancer Genome Atlas (TCGA)-PRAD cohort were obtained from UCSC Xena (http://xena.ucsc.edu/) along with clinical information. The RNA sequencing data, including samples from 499 cases of patients with PRAD and 52 normal samples, were transformed with log2 (count +1) algorithm and then normalized. The median value was taken as the expression value of this gene for a single gene detected by multiple probes. Data of mutation status and immune activity of TCGA-PRAD were accordingly downloaded from UCSC Xena (https://xenabrowser.net/) and Tumor Immunophenotype Profiling (TIP) (http://biocc.hrbmu.edu.cn/TIP/) for further analysis. Similarly, data of an independent dataset from Gene Expression Omnibus (GEO) (GSE116918, https://www.ncbi.nlm.nih.gov/geo/query/acc. cgi?acc=GSE116918) were acquired for necessary external validation [[1](#_ENREF_1)].

**Identification of PRAD potential tumor antigens**

To find the potential tumor antigens of PRAD, the “edgR” R package was first used to identify the upregulated genes in PRAD samples (|log2 (fold change)| > 1 and fold change rate < 0.05) and visualize the location of those genes in chromosomes with Gene Expression Profiling Interactive Analysis (http://gepia2.cancer-pku.cn/#index) [[2](#_ENREF_2)]. “Maftools” R package and cBioportal (https://www.cbioportal.org/) [[3](#_ENREF_3)] was then used to calculate the altered genome fraction and tumor mutational counts in each sample. The intersected part of those upregulated genes and mutated genes were regarded as PRAD potential tumor antigens. The prognostic value of those candidate tumor antigens for OS and DFI were assessed with Cox regression. Genes predictive of both OS and DFI were taken into Tumor Immune Estimation Resource (TIMER) 2.0 database (http://timer.cistrome.org/) [[4](#_ENREF_4)] to explore their correlation with APCs, including B cell, Macrophage, and dendritic cell.

**Identification and validation of the PRAD immune subtypes**

The immunologic signature gene sets were downloaded from The Molecular Signatures Database (MSigDB, https://www.gsea-msigdb.org/gsea/msigdb/) [[5-7](#_ENREF_5)], from which immunologic genes with prognostic significance were found with Lasso regression. The R package “ConsensusClusterPlus” was used to construct the optimal clusters of the TCGA-PRAD samples. The algorithm applied in clustering is partition around medoids (PAM) with a distance metric of “1-Pearson correlation.” The prognostic value of the PRAD immune subtypes in the training cohort was then estimated by Kaplan–Meier survival analysis and validated by the validation cohort. The intra-cohort heterogeneity was calculated by in-group-proportion analysis to confirm the similarity and reproducibility.

**Clinical and cellular features of the PRAD immune subtypes**

Clinical features of the PRAD immune subtypes, including biochemical recurrence (defined as post-treatment prostate-specific antigen serum level > 0.2 ng/mL), pathologic T and N stage, and proportion of patients receiving radiation therapy were displayed using the “ggplot2” R package. More importantly, the SubMap module of GenePattern (https://www.genepattern.org/) was utilized to predict the response of PRAD immune subtypes to immunotherapy. GenePattern can map and merge two different gene expression datasets based on function enrichment, and thus predict the potential phenotypes according to the data of the other dataset without batch effect. Therefore, the PRAD immune subtypes were mapped and merged with the IMvigor210 cohort [[8](#_ENREF_8)], a study of patients with urothelial carcinoma administrating PD-L1 inhibitor atezolizumab, to forecast the possibility of response to anti-PD-L1 treatment. Furthermore, the tumor microenvironment-based ESTIMATE algorithm was applied to calculate the Stromal Score and Immune Score of the PRAD immune subtypes [[9](#_ENREF_9)], and the “CIBERSORT” R package was used to compare the immune cell infiltration among the PRAD immune subtypes.

**Correlation of the PRAD immune subtypes with mutation status and immune status**

Tumor mutation burden (TMB) and mutated gene counts were compared among the PRAD immune subtypes through the “maftools” R package. Copy number variation (CNV) and the mutation of the top 20 mutated genes among tumors were also visualized according to the immune subtypes. Homologous recombination deficiency (HRD) score, including the measurement of telomeric allelic imbalance (named HRD-NtAI), large scale transition (named HRD-LST), and loss of heterozygosity (named HRD-LOH), represents distinct types of genomics scar and chromosomal instability caused by deoxyribonucleic acid repair deficiency and thus is regarded as a powerful biomarker of a given cancer [[10](#_ENREF_10)]. Therefore, the HRD scores for the PRAD immune subtypes were calculated to compare their chromosomal instability with Wilcox test. Besides, mRNA stemness index (mRNAsi), a novel predictor associated with stemlike indices and tumor prognosis [[11](#_ENREF_11)], was also selected to show the difference between the PRAD immune subtypes.

The anticancer immune activity of the PRAD immune subtypes was estimated using the TIP analysis across the seven-step cancer-immunity cycle (http://biocc.hrbmu.edu.cn/TIP/). The correlation of the immune subtypes with immune-checkpoint genes (ICP) and immunogenic cell death-associated genes (ICD) was analyzed with Wilcox test.

**Association between the PRAD immune subtypes and existing PRAD subtypes and biomarkers**

Multiple biomarkers of PRAD have been identified to predict the prognosis of PRAD. Hence, the association constructed between different PRAD biomarkers with the immune subtypes was assessed. In addition, a previous landscape of six immune subtypes, including C1 (Wound Healing), C2 (IFN-γ Dominant), C3 (Inflammatory), C4 (Lymphocyte Depleted), C5 (Immunologically Quiet), and C6 (TGF-β Dominant) for pan-cancer, has been published and was compared with our PRAD immune subtypes [[12](#_ENREF_12)].

**Immune landscape and** **weighted correlation network analysis (WGCNA) of PRAD**

The immune landscape of PRAD is analyzed with the “monocle” R package to perform the trajectory plotting of each sample. The discriminative dimensionality reduction with trees was used to visualize the trajectory of immune subtypes with maximal number components of 2. For immune subtypes distributed in different branches of the tree, principal components analysis was performed to further analyze the immune subtype distribution.

The R package “WGCNA” was selected to construct the weighted correlation network of immune-associated genes (power = 9), and different modules of the network were acquired. Eigengene of each module was calculated, and modules with prognostic value of OS were accordingly identified (*P* < 0.05). The correlation between genes within the prognostic modules and eigengenes were calculated, and genes with a correlation >0.9 and a *P* value of <0.05 in multivariate Cox regression were identified to construct the risk model, for which *exp* means the gene expression value, *i* means each sample, *j* means each gene, and *β* means the coefficient in multivariate regression:

RiskScore_i_ = $\sum_{j=1}^{n} {Exp}_{ji}\times\beta_{j}$

**Differential expression gene (DEG)-based risk model construction**

DEG among the PRAD immune subtypes was identified and then put into univariate Cox regression to find the prognostic DEGs. Lasso regression was used to reduce dimension and constructed a risk model to discriminate patients into high-risk and low-risk groups. The risk score of different immune subtypes and the distribution of immune subtypes in high-risk and low-risk groups were also plotted.

**References**

1. Jain S, Lyons CA, Walker SM, McQuaid S, Hynes SO, Mitchell DM, Pang B, Logan GE, McCavigan AM, O'Rourke D, et al: **Validation of a Metastatic Assay using biopsies to improve risk stratification in patients with prostate cancer treated with radical radiation therapy.** *Ann Oncol* 2018, **29:**215-222.

2. Tang Z, Li C, Kang B, Gao G, Li C, Zhang Z: **GEPIA: a web server for cancer and normal gene expression profiling and interactive analyses.** *Nucleic Acids Res* 2017, **45:**W98-w102.

3. Cerami E, Gao J, Dogrusoz U, Gross BE, Sumer SO, Aksoy BA, Jacobsen A, Byrne CJ, Heuer ML, Larsson E, et al: **The cBio cancer genomics portal: an open platform for exploring multidimensional cancer genomics data.** *Cancer Discov* 2012, **2:**401-404.

4. Li T, Fu J, Zeng Z, Cohen D, Li J, Chen Q, Li B, Liu XS: **TIMER2.0 for analysis of tumor-infiltrating immune cells.** *Nucleic Acids Res* 2020, **48:**W509-w514.

5. Subramanian A, Tamayo P, Mootha VK, Mukherjee S, Ebert BL, Gillette MA, Paulovich A, Pomeroy SL, Golub TR, Lander ES, Mesirov JP: **Gene set enrichment analysis: a knowledge-based approach for interpreting genome-wide expression profiles.** *Proc Natl Acad Sci U S A* 2005, **102:**15545-15550.

6. Godec J, Tan Y, Liberzon A, Tamayo P, Bhattacharya S, Butte AJ, Mesirov JP, Haining WN: **Compendium of Immune Signatures Identifies Conserved and Species-Specific Biology in Response to Inflammation.** *Immunity* 2016, **44:**194-206.

7. Liberzon A, Subramanian A, Pinchback R, Thorvaldsdóttir H, Tamayo P, Mesirov JP: **Molecular signatures database (MSigDB) 3.0.** *Bioinformatics* 2011, **27:**1739-1740.

8. Balar AV, Galsky MD, Rosenberg JE, Powles T, Petrylak DP, Bellmunt J, Loriot Y, Necchi A, Hoffman-Censits J, Perez-Gracia JL, et al: **Atezolizumab as first-line treatment in cisplatin-ineligible patients with locally advanced and metastatic urothelial carcinoma: a single-arm, multicentre, phase 2 trial.** *Lancet* 2017, **389:**67-76.

9. Yoshihara K, Shahmoradgoli M, Martínez E, Vegesna R, Kim H, Torres-Garcia W, Treviño V, Shen H, Laird PW, Levine DA, et al: **Inferring tumour purity and stromal and immune cell admixture from expression data.** *Nat Commun* 2013, **4:**2612.

10. Marquard AM, Eklund AC, Joshi T, Krzystanek M, Favero F, Wang ZC, Richardson AL, Silver DP, Szallasi Z, Birkbak NJ: **Pan-cancer analysis of genomic scar signatures associated with homologous recombination deficiency suggests novel indications for existing cancer drugs.** *Biomark Res* 2015, **3:**9.

11. Zhang C, Chen T: **Depiction of tumor stemlike features and underlying relationships with hazard immune infiltrations based on large prostate cancer cohorts.** 2021, **22**.

12. Thorsson V, Gibbs DL, Brown SD, Wolf D, Bortone DS, Ou Yang TH, Porta-Pardo E, Gao GF, Plaisier CL, Eddy JA, et al: **The Immune Landscape of Cancer.** *Immunity* 2019, **51:**411-412.
